# Exosomes exert cardioprotection in dystrophin-deficient cardiomyocytes via ERK1/2-p38/MAPK signaling

**DOI:** 10.1038/s41598-018-34879-6

**Published:** 2018-11-08

**Authors:** Melanie Gartz, Ashley Darlington, Muhammed Zeeshan Afzal, Jennifer L. Strande

**Affiliations:** 1 Cardiovascular Center, Medical College of Wisconsin, Milwaukee, WI USA; 2 Department of Medicine, Cardiovascular Medicine, Medical College of Wisconsin, Milwaukee, WI USA; 3 Department of Cell Biology, Neurobiology, and Anatomy, Medical College of Wisconsin, Milwaukee, USA

## Abstract

As mediators of intercellular communication, exosomes containing molecular cargo are secreted by cells and taken up by recipient cells to influence cellular phenotype and function. Here we have investigated the effects of exosomes in dystrophin-deficient (Dys) induced pluripotent stem cell derived cardiomyocytes (iCMs). Our data demonstrate that exosomes secreted from either wild type (WT) or Dys-iCMs protect the Dys-iCM from stress-induced injury by decreasing reactive oxygen species and delaying mitochondrial permeability transition pore opening to maintain the mitochondrial membrane potential and decrease cell death. The protective effects of exosomes were dependent on the presence of exosomal surface proteins and activation of ERK1/2 and p38 MAPK signaling. Based on our findings, the acute effects of exosomes on recipient cells can be initiated from exosome membrane proteins and not necessarily their internal cargo.

## Introduction

Dystrophin-deficiency results from mutations in the *DMD* gene and can manifest as Duchenne muscular dystrophy (DMD). DMD is characterized by severe limb and diaphragm muscle weakness in which patients lose independent ambulation and develop respiratory weakness within the first and second decades of life, respectively^1^. Cardiomyopathy occurs within the second to third decade and is consequently a leading cause of death for these patients^2^.

The dystrophin protein functions to stabilize the sarcolemma during muscle contraction and relaxation by linking the actin cytoskeleton to the extracellular matrix via the dystrophin-glycoprotein complex (DGC)^3^. The absence of dystrophin destabilizes the DGC, altering stress-induced intracellular signaling as evidenced by membrane rupture^4^, increased intracellular calcium^5,6^ dysregulated NO^7^, increased reactive oxygen species (ROS)^6,8^, and mitochondrial dysfunction^9^. In addition to intracellular signaling, intercellular signaling may to be perturbed^10^.

Extracellular vesicles such as exosomes serve as a mode of intercellular communication by transferring their cargo consisting of mRNAs, microRNAs (miRs), lipids and proteins from one cell to another to influence cellular phenotypes^11,12^. In skeletal muscle, dystrophin-deficiency leads to dysregulation of vesicle trafficking along with the disturbance of cargo proteins and microRNAs^13,14^. However, the functional effects of these dysregulated exosomes are largely unknown. The potential of exosomes to mediate cardiac repair in the ischemic myocardium has been well established and there is developing evidence that exosomes may also benefit dilated cardiomyopathies such as dystrophin-deficient cardiomyopathy^15^. However, most of these beneficial effects occur from the application of stem cell or progenitor cell secreted exosomes. For example, exosomes derived from mesenchymal stem cells, cardiac progenitor cells, and hematopoietic stem cells promote angiogenesis, decrease apoptosis, and improve cardiac function in experimental myocardial ischemia^16–19^. Exosomes secreted from cardiosphere-derived progenitor cells have been shown to mediate an improvement in cardiac function in the dystrophin-deficient (*mdx*) mouse with associated evidence showing the exosomes may be inducing the anti-oxidative pathway, enhancing the mitochondrial biogenesis signaling pathway, and decreasing apoptosis^15^. However, considering exosomes are secreted by a variety of cell types in the heart including smooth muscle cells, endothelial cells, fibroblasts, immune cells and cardiomyocytes^20^, little is known about endogenous exosome modulation of the cardiomyocyte phenotype in the dystrophin-deficient heart. Considering that endogenous exosomes secreted in the dystrophin-deficient skeletal muscle have been shown to contribute to muscle fibrosis in DMD^10^ and that exosomes derived from diseased cardiomyocytes are known to be pathogenic and have adverse effects on neighboring cardiomyocytes^21,22^, we postulated that dystrophin-deficient-cardiomyocyte exosomes would be detrimental in the dystrophin-deficient heart.

Extracellular spaces in the heart would have a mixture of exosomes from different cell sources making it difficult to distinguish cell-specific exosome effects on cardiomyocytes. Therefore, in this study, we used two unrelated patient-specific induced pluripotent stem cell (iPSC) lines containing DMD exon 3–6 deletion mutations and a gene-edited iPSC line in which the DMD exon 1 was targeted by CRISPR/Cas9. We have previously reported that dystrophin-deficient (Dys) iPSC- derived cardiomyocytes (Dys-iCMs) are exceptionally susceptible to the effects of cellular stress by increasing ROS, mitochondrial dysfunction and undergoing apoptosis^23^. Understanding how endogenous exosomes are changing the phenotype of the dystrophin-deficient cardiomyocyte will give greater understanding of the mechanisms of dystrophin-deficient cardiomyopathy.

## Results

### Generation and characterization of Dys-iPSCs and derived cardiomyocytes

To assess the intercellular signaling between cardiomyocytes, we used two DMD patient derived iPSC lines and one gene-edited iPSC in which the DMD gene was targeted by CRISPR/Cas9. The Dys1-iPSC line derived iCMs contain an DMD exon 3–6 deletion have been previously described and characterized^23^. The patient-derived Dys3-iPSC line was generated by reprogramming urine progenitor cells from a DMD patient harboring a DMD exon 3–6 mutation (Supplemental Fig. 1). The WT2-iPSC line was used to create the DysC-iPSC line by CRISPR/Cas9 targeting of DMD exon 1 to create a 6 bp deletion leading to dystrophin deficiency (Supplemental Fig. 2). WT1 and WT2-iCMs were included as non-disease controls. All iPSC lines were able to differentiate into cardiomyocytes and displayed typical cardiogenic markers^23^ (Supplemental Fig. 3).

### Dys-iCMs secrete paracrine signals that are protective for Dys-iCMs but not WT-iCMs

**Figure 1:**
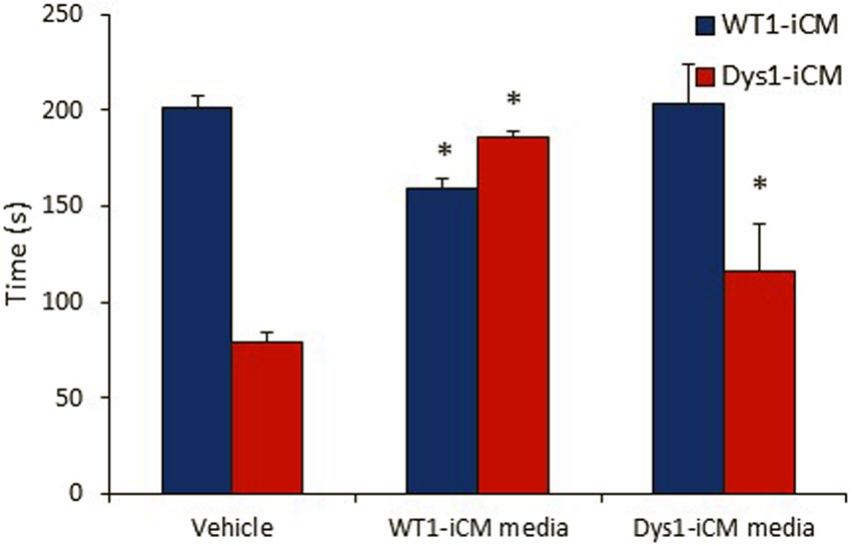
Treating Dys1-iCM with conditioned media delays opening of mPTP. Conditioned media collected from cardiomyocytes and added 2 hours prior to inducing mPTP formation with laser scanning confocal microscopy. WT1-iCM media, but not Dys1-iCM media delayed mPTP in WT1-iCMs. Both WT1- and Dys1-iCM media delayed mPTP formation in Dys1-iCMs. *p < 0.05 vs. vehicle, n = 3/group.

We first used a mitochondrial permeability transition pore (mPTP) opening assay as a functional screen to determine whether conditioned media had paracrine effects on WT1 and Dys1-iCM function. We have previously shown that Dys1-iCMs open the mPTP earlier than WT1-iCMs^23^ and confirmed this phenotype in Fig. 1. Conditioned media from both WT1- and Dys1-iCMs, when applied to Dys1-iCMs, significantly delayed mPTP opening time compared to the vehicle group. Conditioned media from Dys1-iCMs did not change mPTP opening time in WT1-iCMs and conditioned media from WT1-iCMs delayed mPTP opening time in WT1-iCMs. This suggested differential paracrine effects between the WT1 and Dys1-iCM conditioned media.

### Characterization of extracellular vesicles secreted from WT and Dys-iCMs

**Figure 2:**
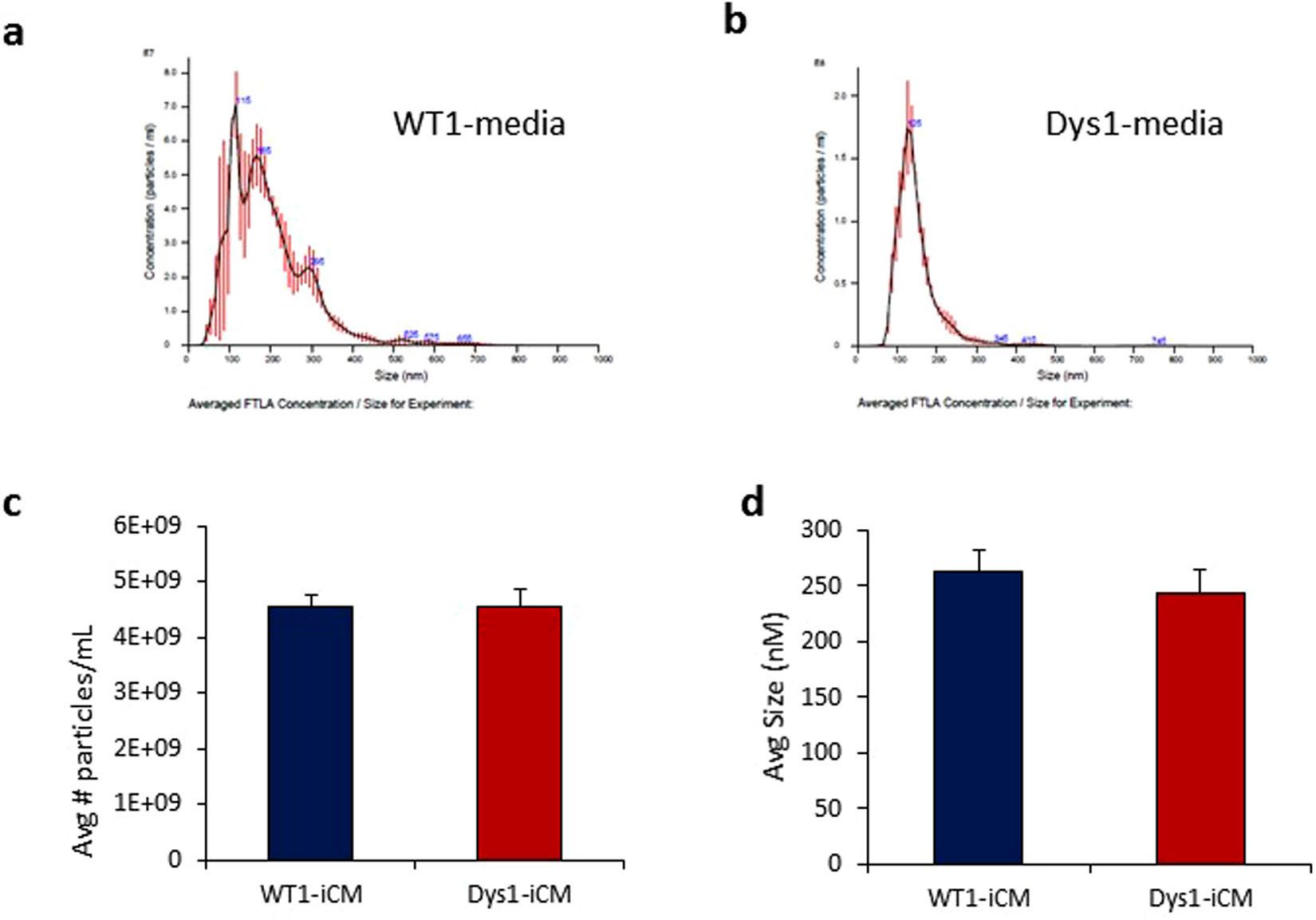
Nanoparticle tracking analysis (NTA) showing the variety of extracellular vesicles present in the conditioned media. Representative NTA profiles from (**a**) WT1-iCM conditioned media and (**b**) Dys1-iCM conditioned media. Both WT1- and Dys1-iCMs secreted (**c**) similar numbers and (**d**) similar sized extracellular vesicles. n = 3/group. (n.s.)

iCM conditioned media was analyzed by nanoparticle tracking analysis (NTA) to detect and characterize iPSC cardiomyocyte-secreted extracellular vesicles (Fig. 2a,b). Both WT1 and Dys-iCMs secreted similar numbers of extracellular vesicles (Fig. 2c) and although the Dys1-iCMs secreted slightly smaller (average 243 nm, range 50–600 nm) sized vesicles than WT1-iCMs (average 263 nm, range 25–600 nm), this difference was not significant (Fig. 2d).

**Figure 3:**
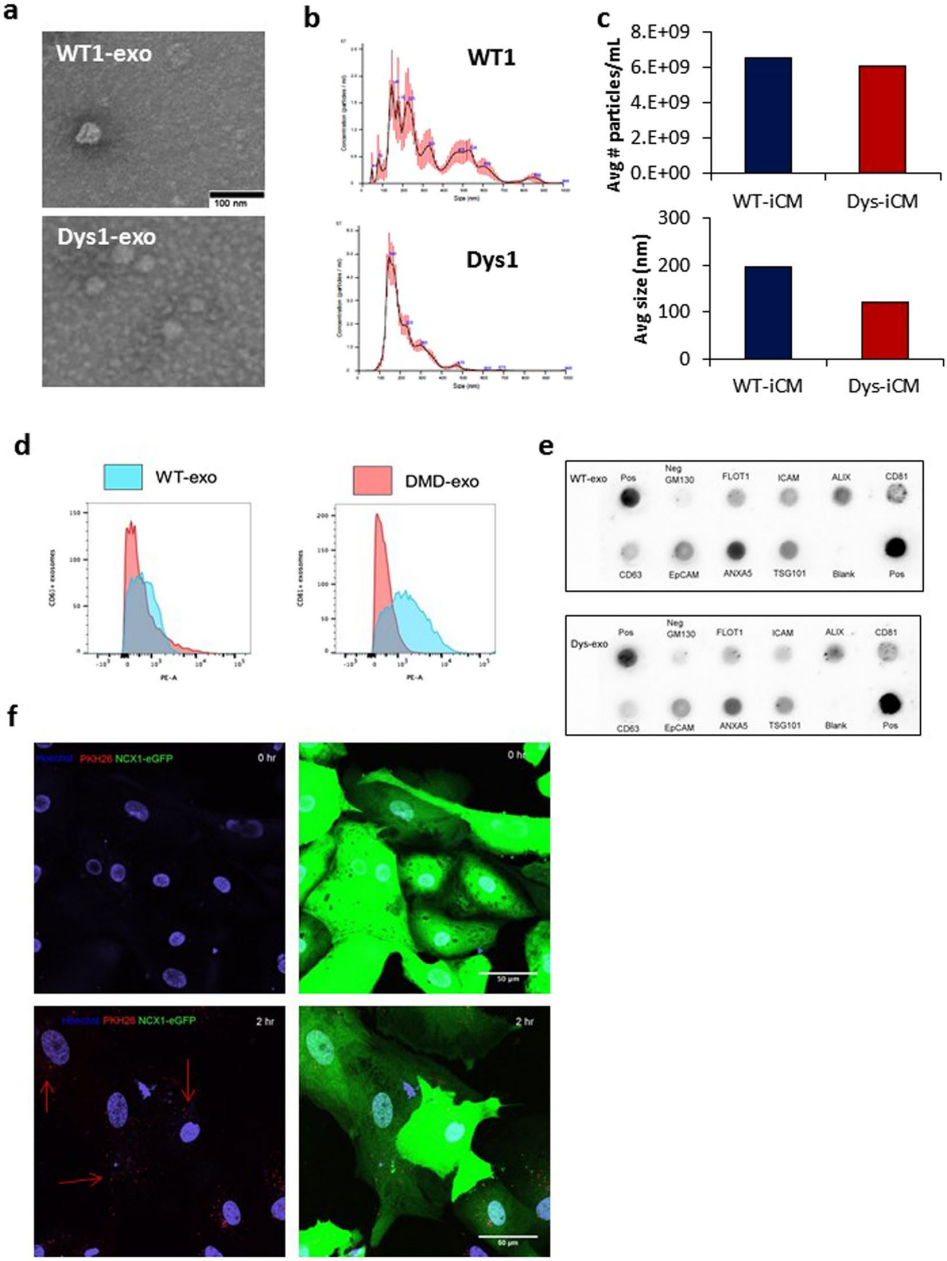
Characterization of isolated exosomes. (**a**) Electron microscopy images reveal WT- and Dys-iCM secreted exosomes display traditional cuplike morphology and are approximately 50 nm. (**b**) NTA of isolated exosomes reveals a range of sizes in particles averaging 148 nm (WT-exo) and 187 (Dys-exo). (**c)** Quantitation of NTA results. (**d)** Exosomes exhibit exosomal markers CD63 and CD81 as shown by flow cytometry. (**e**) Exo-Check protein array analysis reveals WT- and Dys-exo display exosome protein markers. (**f**) Cardiomyocytes are labeled with NCX1-eGFP and exosomes are stained with PKH26 (red). Exosomes are seen to be taken up at 2 hours.

We then characterized exosomes isolated from the conditioned media of WT1 and Dys1-iCMs. NTA and transmission electron microscopy revealed that WT1-iCM and Dys1-iCM exosomes (WT-exos and Dys-exos) were as small as 50 nm in diameter (Fig. 3a,b), but averaged 193 and 148 nm, respectively (Fig. 3c). Flow cytometric analysis of exosome-coated latex beads confirmed the presence of conventional exosome membrane markers, particularly CD63 and CD81 (Fig. 3d). The unlabeled exosome-coated beads without the primary antibody +/− the secondary antibody did not fluorescence (Supplemental Fig. 4). Furthermore, a surface protein array confirmed the presence of FLOT1, ICAM, ALIX, EpCAM, ANXA5, TSG101 on the surface of both WT-exos and Dys-exos (Fig. 3e). Using confocal microscopy, we show that iCMs successfully took up PKH26-labeled exosomes by 2 hours (Fig. 3f). Supplemental Video 1 shows Z-stack imaging of cardiomyocytes with labeled exosomes distributed throughout the cytoplasm and nucleus.

### Cardiomyocyte-exosomes protect against stress-induced injury in Dys-iCMs

We then investigated whether WT-exos and Dys-exos were responsible for the functional effects of conditioned media. Previously, it has been shown that Dys-iCMs were especially vulnerable to cellular stress by increasing ROS levels to increase cell death^23,24^. Therefore, we used ROS levels and cell death as endpoints when conducting a dose concentration curve. Five µL containing approximately 2.25 × 10^7^ exosomes displayed the optimal cardioprotective properties in Dys-iCMs (Supplemental Fig. 5). This dose was used for all further studies.

**Figure 4:**
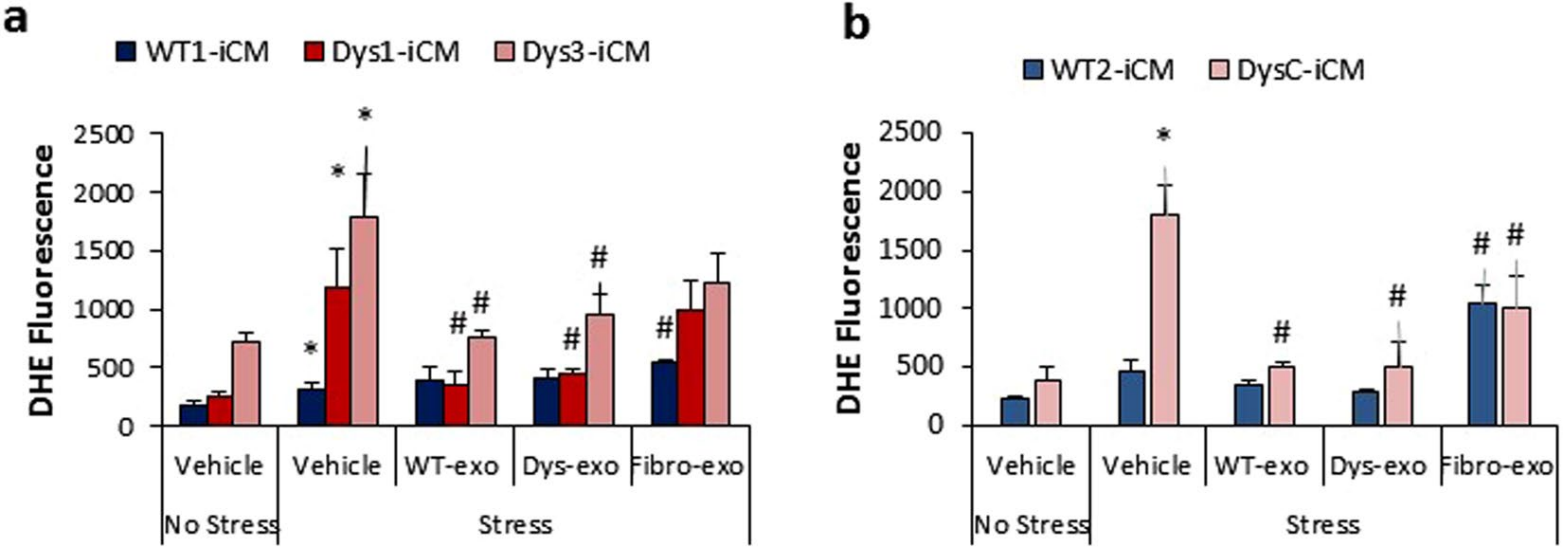
Exosomes protect against stress-induced cell injury in Dys-iCMs. Exposure to WT- and Dys-exos prior to stress (**a**,**b**) decreased ROS levels. Dermal fibroblast exos did not reduce stress induced ROS levels in Dys1-iCMs as significantly as iCM-derived exos. n = 3/group, *p < 0.05 vehicle stress vs. vehicle no stress, ^#^p < 0.05 exosome exposure vs. vehicle stress.

Exosomes acutely decreased injury-induced ROS levels in all Dys-iCMs compared to vehicle control (Fig. 4a,b). Exosomes isolated from the conditioned media of dermal fibroblasts (fibro-exos) were used as an inert exosome control^25^. However, fibro-exos were not consistently inert showing some ability to protect the iCMs against stress-induced ROS, but not as significantly as cardiomyocyte exosomes, suggesting that the cardioprotective paracrine signaling was enhanced in cardiomyocyte exosomes.

### Cardiomyocyte exosomes protect against mitochondrial triggers of apoptosis in Dys-iCMs

**Figure 5:**
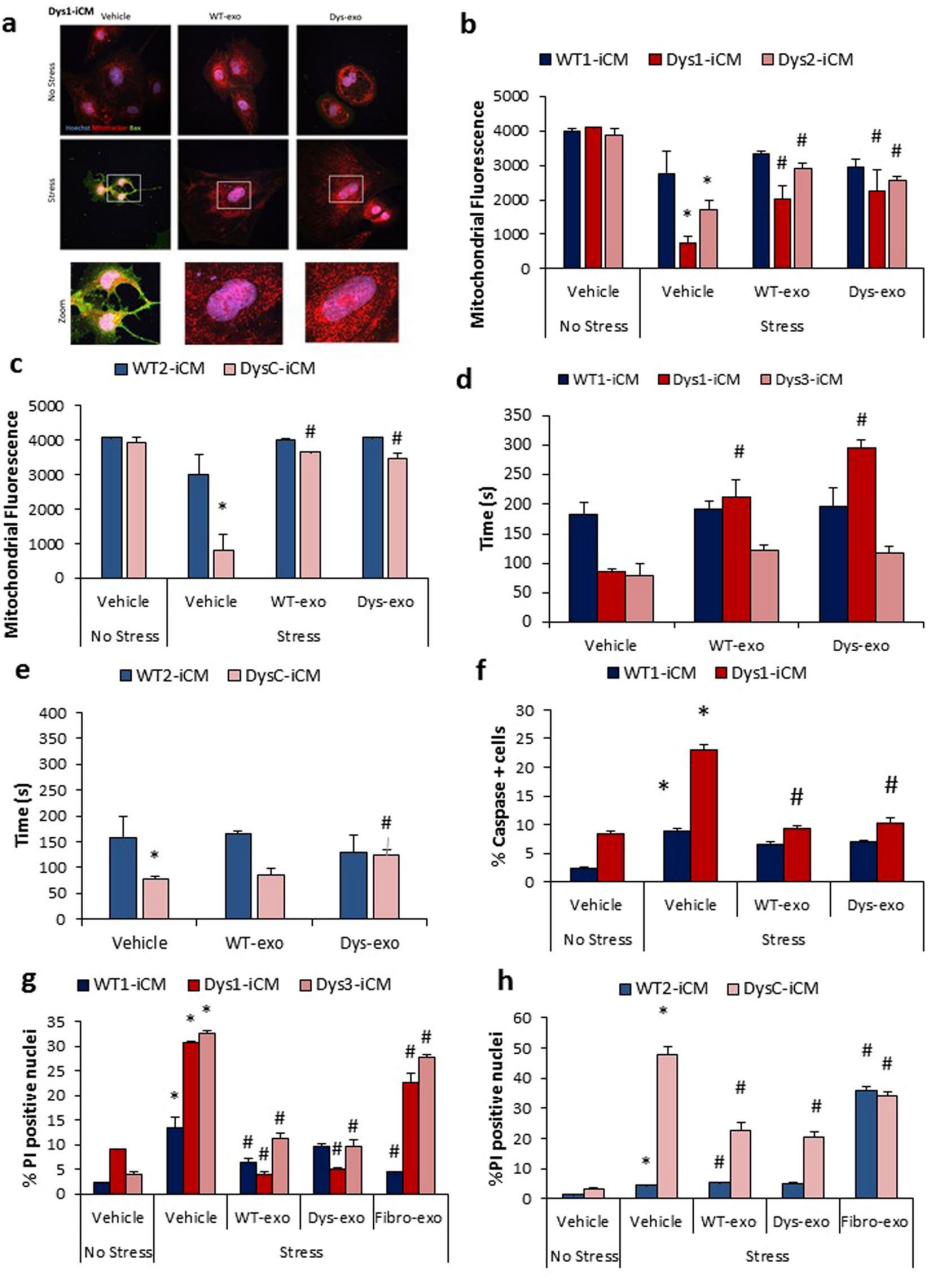
Exosomes protect against mitochondrial triggers of apoptosis in Dys-iCMs. (**a**) Dys1-iCM mitochondria displayed higher levels of Bax (green) positive mitochondria (Mitotracker, red) following stress which was ameliorated following exosome exposure. Exosome exposure also (**b**,**c)** prevented stress-induced loss of mitochondrial membrane potential; (**d**,**e**) delayed mPTP formation, (**f)** decreased % caspase positive cells; and (**g**,**h**) mitigated stress-induced increases in PI positive nuclei. n = 3/group. *p < 0.05 vehicle stress vs. vehicle no stress, ^#^p < 0.05 exosome exposure vs. vehicle stress.

We next investigated whether WT- and Dys-exos could mitigate stress-induced mitochondrial apoptotic pathways. Bax translocation to the mitochondria, with the loss of the mitochondrial membrane potential, can trigger opening of the mitochondrial permeability transition pore followed by caspase activation and cell death^26^. WT- and Dys-exos inhibited Bax expression and mitochondrial translocation in Dys1-iCMs in contrast to vehicle treated Dys-iCMs subject to stress (Fig. 5a). Stress does not cause Bax translocation in WT1-iCMs (Supplemental Fig. 6). Stress induces a loss of mitochondrial membrane potential (Fig. 5b,c) and an earlier mPTP opening time (Fig. 5d,e) in Dys1, Dys3 and DysC-iCMs, all of which is improved following exosome exposure and in comparison, to WT1 and WT2-iCMs. Both WT- and Dys-exos decreased the levels of caspases 3/7 in Dys1-iCMs (Fig. 5f) but had no effects on WT1-iCMs. Both WT- and Dys-exos decrease stress-induced cell death as detected by propidium iodide staining in all Dys-iCMs (Fig. 5g,h). Fibro-exos mildly decreased cell death in Dys1, Dys3, DysC and WT1-iCMs but not to the same extent seen with cardiomyocyte exos. Interestingly, fibro-exos exacerbated stress-induced cell death in WT2-iCMs (Fig. 5h).

In summary, these data reveal that both WT- and Dys-exosomes decrease translocation of Bax to the mitochondria, preserve the mitochondrial membrane potential, delay the mPTP opening time, and decrease caspase 3/7 activity along with cell death. Dys-iCMs are more susceptible to the protective effects of both WT- and Dys-exos, whereas Dys-exos do not completely protect WT-iCMs against stress.

### Cardioprotective effects of cardiomyocyte exosomes depend on the ERK1/2 and p38 MAPK signaling pathway

**Figure 6:**
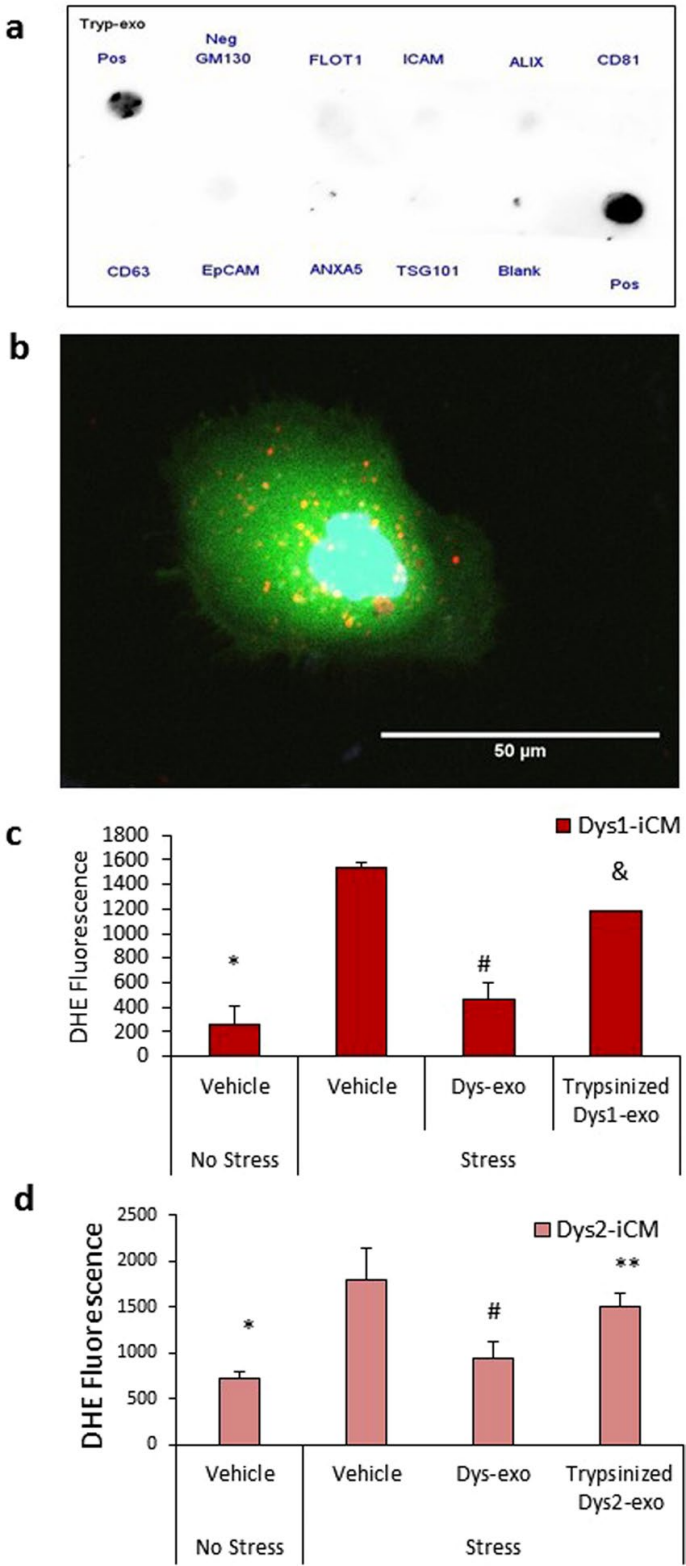
Surface proteins are required for exosomes to stimulate cardioprotection in Dys-iCMs. **(a**) Trypsinized Dys1-exos do not display exosomal surface markers, and (**b**) are intact and readily taken up into iCMs at 2 hr. (**c)** Exposure of Dys1-iCMs and (**d)** Dys3-iCMs to trypsinized exos failed to reduce ROS levels as significantly as intact exos. n = 3/group. *p < 0.05 vehicle stress vs. vehicle no stress, ^#^p < 0.05 exosome exosome vs. vehicle stress, ^&^p < 0.05 Trypsinized exosomes vs. intact exosomes, **p = 0.07 Trypsinized exosomes vs. intact exosomes.

Exosomal transfer of microRNA has been shown to protect cardiomyocytes by controlling cell survival gene expression^27^. However, considering the iCMs were exposed to exosomes for only 2 hours, we did not expect gene expression changes, but rather surmised exosomes would exert protective effects through an exosome surface ligand to activate cardiomyocyte cell survival pathways. To investigate whether an exosomal surface protein was required for its cardioprotective properties, we cleaved the surface proteins from Dys-exos with trypsin. Protein array analysis confirmed the absence of exosome membrane proteins after trypsin digestion (Fig. 6a). Trypsin-treated exosomes labeled with PKH26 were intact and taken up into iCMs at 2 hr (Fig. 6b). When compared to the intact exosomes, the trypsin-treated exosomes did not decrease stress-induced ROS levels in Dys1 or Dys3-iCMs (Fig. 6c,d), indicating a role for exosomal surface proteins in triggering the protective effect of exosomes.

**Figure 7:**
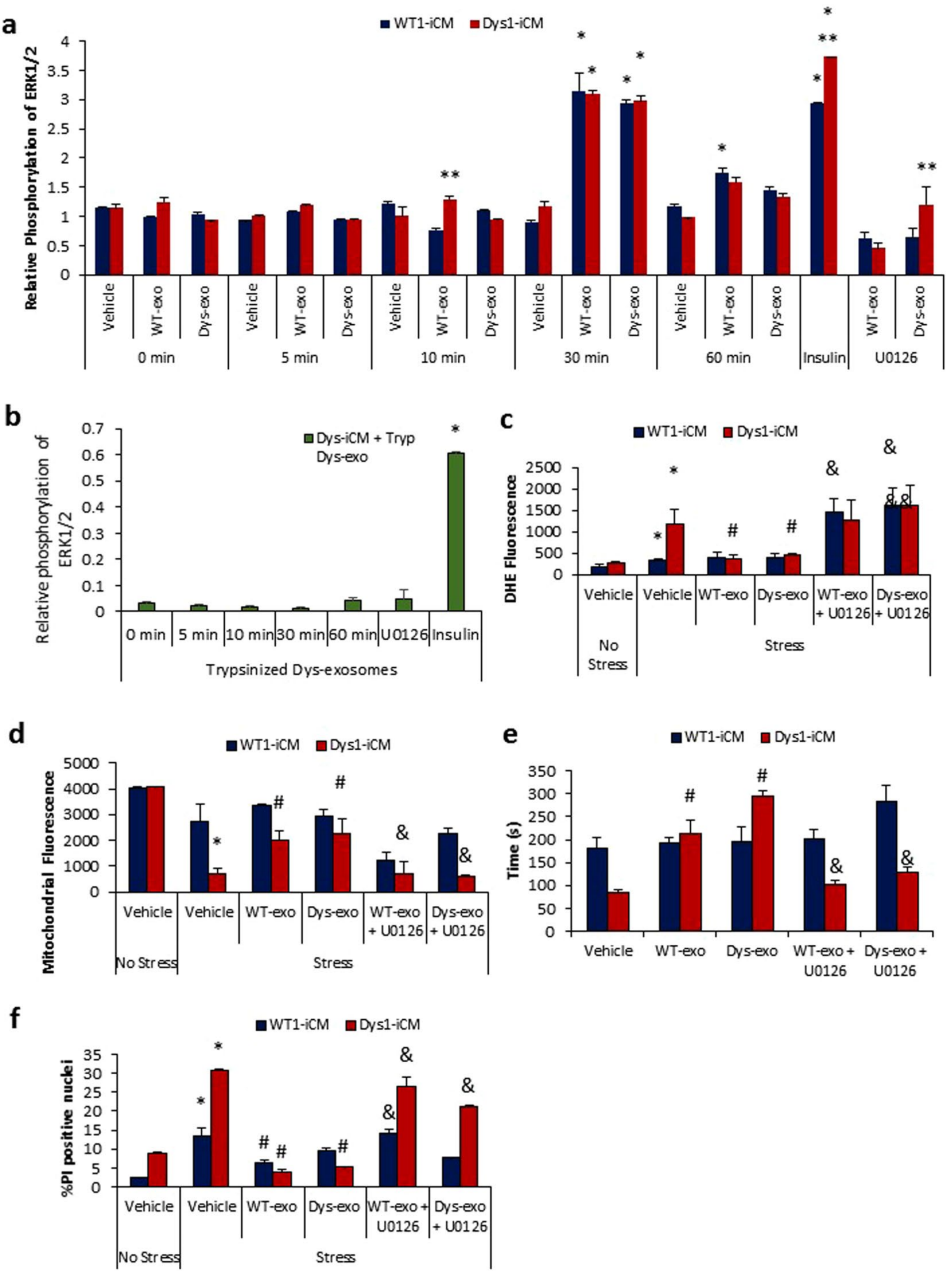
Cardioprotective effects of cardiomyocyte-exosomes depend on ERK1/2. (**a**) Analysis of relative phosphorylation reveals that exosome exposure in WT1 and Dys1-iCMs stimulates phospho-ERK1/2 at 30 minutes. n = 9–12/group, *p < 0.05 vs. 0 min. vehicle, **p < 0.05 Dys-iCM vs. WT-iCM. (**b)** Trypsinized exosomes failed to stimulate ERK1/2 phosphorylation. Blocking ERK1/2 with U0126 prevented protection leading to (**c**) increased ROS levels, (**d)** dissipation of membrane potential, (**e**) early mPTP formation, and (**f**) increased cell death. n = 3/group. *p < 0.05 vehicle stress vs. vehicle no stress, ^#^p < 0.05 exosome exposure vs. vehicle stress, ^&^p < 0.05 inhibitor vs. exosome exposure.

The mitogen-activated protein kinases (MAPK) including ERK1/2 and p38 MAPK coordinate responses that dictate cell death or survival^28^. ERK1/2 phosphorylation was stimulated at 30 min after exposure to both WT and Dys-exos (Fig. 7a). Trypsinized exosomes failed to stimulate ERK1/2 phosphorylation (Fig. 7b). U0126, a specific inhibitor of MEK1, was used to examine the potential role of the ERK1/2 pathway in WT and Dys-exos cardioprotection of iCMs. ERK1/2 inhibition abolished the protective effects of WT and Dys-exos on stress-induced ROS increases in Dys1-iCMs (Fig. 7c), mitochondrial membrane potential (Fig. 7d), mPTP formation (Fig. 7e) and cell death (Fig. 7f). ERK1/2 inhibition alone with U0126 exacerbated the stress-induced increase in cell death (Supplemental Fig. 7) implicating its anti-apoptotic role in Dys-iCMs.

U0126 is known to block exosome uptake by disrupting lipid raft-mediated endocytosis^29,30^, therefore, we examined whether U0126 was inhibiting cardioprotection by blocking cellular uptake of exosomes. Supplemental Fig. 7 and Supplemental Video 2 show that cells pretreated with U0126 prior to the addition of PKH26-labeled exosomes were readily taking up the exosomes by 2 hours. This suggests that U0126 at this specific concentration did not impede exosome uptake, and therefore the reversal of the cardioprotective effect observed was likely due to the absence of an exosome surface protein to initiate ERK1/2 pathway activation.

**Figure 8:**
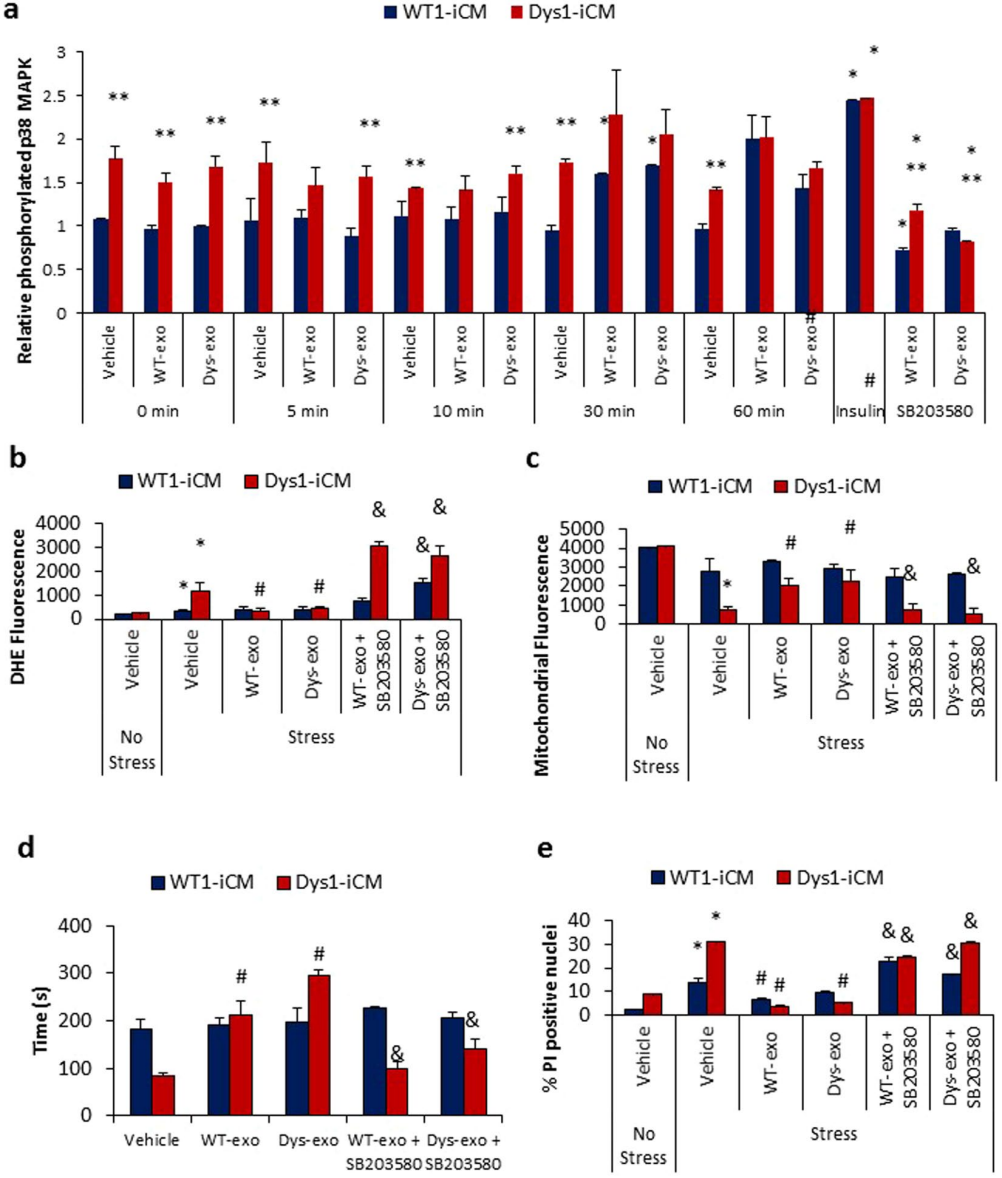
Cardioprotective effects of cardiomyocyte-exosomes depend on p38/MAPK. (**a**) Analysis of relative phosphorylation in WT1 and Dys1-iCMs reveals that exosome exposure stimulates phosphorylation of p38 MAPK at 30 minutes. n = 9–12/group, *p < 0.05 vs. 0 min. vehicle, **p < 0.05 Dys-iCM vs. WT-iCM, ^#^p = 0.3 vs. vehicle. Blocking p38/MAPK with SB203580 in Dys1-iCMs was associated with (**b)** increased ROS levels, (**c)** a decrease in mitochondrial membrane potential, (**d)** early mPTP formation and (**e)** increased cell death. n = 3/group. *p < 0.05 vehicle stress vs. vehicle no stress, ^#^p < 0.05 exosome exposure vs. vehicle stress, ^&^p < 0.05 inhibitor vs. exosome exposure.

We next examined the involvement of p38 MAPK with respect to the cardioprotective effects of exosomes. The basal phosphorylation of p38 MAPK was increased in Dys-iCMs compared to WT-iCMs (Fig. 8a). WT and Dys-exos stimulated phosphorylation of p38 MAPK at 30 min post exposure (Fig. 8a). Inhibiting p38 MAPK with SB203580 not only reversed the protective effects WT-exos and Dys-exos on Dys-iCMs but also exacerbated the stress-induced increase of ROS (Fig. 8b). SB203580 reversed the protective effects of WT- and Dys-exos on stress-induced loss of the mitochondrial membrane potential (Fig. 8c), mPTP opening (Fig. 8d) and cell death (Fig. 8e). Inhibition of p38 MAPK alone further increased stress-induced ROS levels and cell death when compared vehicle treated groups (Supplemental Fig. 8).

## Discussion

The present study was conceptualized to determine the paracrine effects of exosomes secreted from dystrophin-deficient cardiomyocytes on dystrophin-deficient cardiomyocytes. We initially postulated that Dys-exos would be detrimental to the functional effects on Dys-cardiomyocytes by exacerbating stress-induced cell injury. *In vivo*, the concentration or density exosomes in the intercellular space is unknown. Therefore, we started with a concentration-response curve to assess the functional effects of exosomes on Dys-iCMs. Unexpectedly, we found both WT- and Dys-exos were protective against stress-induced injury in the Dys-iCM rather than being detrimental. Therefore, we continued to investigate the mechanism downstream of the cardioprotective properties of exosomes.

We show that exosomes secreted from either WT- or Dys-iCMs protect the Dys-iCMs against stress induced increases in ROS levels, decreases in the mitochondrial membrane potential, and cell death. We have confirmed these findings in two unrelated DMD patient-specific iCMs (Dys1 and Dys3) along with a DMD gene-edited iCM lines (DysC) suggesting this is a robust effect of cardiomyocyte-specific exosomes on Dys-iCMs independent of iPSC clonal or genetic variation between iPSC lines. In addition, we provide supporting evidence that iCM-derived exosomes decrease Bax translocation to the mitochondria and caspase activation. Cardiomyocyte-derived exosomal cardioprotective effects were not replicated using exosomes secreted from dermal fibroblasts suggesting that cardiomyocyte-derived exosomes are enriched in cardioprotective factors.

Dermal fibroblast exosomes were previously shown to be an inert control for cardioprotection due to differences in microRNA cargo^16,25^ compared to exosomes secreted from cultured stem cells and progenitor cells *in vitro* which are cardioprotective in *in vitro* and *in vivo* models of myocardial ischemia^18,31,32^. However, these studies focus on the transfer cargo inside the cell, mainly microRNA delivered by the exosome to alter gene expression to change the phenotype of the cardiomyocyte. In our study, we provide evidence that endogenous exosomes from dystrophin-deficient cardiomyocytes depend on a surface protein to retain its cardioprotective properties on the dystrophin-deficient cardiomyocyte suggesting that an exosomal surface protein may be involved in its paracrine effects. PKH26-labeled labelled exosomes taken up by the iCMs within the 2-hour exposure does not eliminate the possibility that microRNAs are contributing to the cardioprotective phenotype. However, cleaving the exosome surface proteins with trypsin and inhibiting ERK1/2 and p38 MAPK signaling pathways abrogates the exosome-mediated cardioprotection, which strongly suggests that these effects are mediated by a ligand-receptor interaction. However, we do not know whether this interaction occurs at the sarcolemma of the cardiomyocyte or after internalization of the exosomes. Exosomes are known to induce the phosphorylation of several downstream targets including p38 MAPK and ERK1/2^29,33,34^. Both activation of ERK1/2 and p38 MAPK signaling pathways are known to be acutely cardioprotective against cardiac stress by through anti-apoptotic mechanisms^35^. Both ERK1/2 and p38 MAPK have been demonstrated to form signaling modules by inhibiting GSK-3β^36^ at the level of the mitochondria^37^ to inhibit mPTP opening^38^. Both ERK1/2 and p38 MAPK have the ability to activate heat shock protein 27 which has previously been shown to play a role in exosome mediated protection of myocardial ischemia^39^.

It has been shown that ERK1/2 is activated during lipid-raft dependent exosome uptake^29^. Specifically, the ERK1/2 inhibitor U0126 has been shown decrease exosome uptake in a dose dependent manner. In our study, the concentration of U0126 used blocks ERK1/2 phosphorylation and mitigates the cardioprotective effect of exosomes but does not block exosome uptake in the iCMs. Therefore, we do not know whether this interaction occurs at the sarcolemma of the cardiomyocyte or after internalization of the exosomes.

A surface protein on the exosomes appeared to be involved in triggering acute protective signaling, as cleaving off these proteins with trypsin negated the cytoprotection of Dys-iCMs. For the first time, in this study, we provide evidence that exosome treatment stimulates cardioprotective signaling pathways in an *in vitro* model of dystrophin-deficient cardiomyopathy. This is a novel approach to understanding the mechanisms involved in this unique cardiomyopathy, and offers a way to further that understanding, as well as to investigate future therapies. Identifying the exosomal surface protein involved in initiating the cardioprotective effects may provide a novel target for the treatment of dystrophin-deficient cardiomyopathy. Studies to identify this protein are underway.

In summary, this study demonstrates that acute exposure of endogenous cardiomyocyte-secreted exosomes has the potential to protect against cellular stress in dystrophin-deficient cardiomyocytes. The protective pathways that are stimulated in this process include ERK1/2 and p38 MAPK. These signaling pathways are triggered by a surface receptor that is present on myocyte-secreted exosomes. Our results indicate that these pathways may be dysregulated in dystrophin-deficient cardiomyopathy, and offer a therapeutic target of interest in acute protection of the dystrophin-deficient heart against stress-induced injury.

## Methods

### Cell Lines and Cellular Reprogramming

For this study, we used previously characterized induced pluripotent stem cell (iPSC) lines: a Dys1-iPSC line which contains an out-of-frame dystrophin gene deletion of exons 3–6 resulting in a null mutation with the complete absence of the dystrophin protein (SC604A/B-MD, Systems Biosciences; Mountain View, CA), a non-dystrophic wild-type iPSC line (WT1-iPSC) which was a generous gift from Dr. April Pyle^40^. A second dystrophin-deficient line (Dys3-iPSC) with dystrophin null mutation resulting from deletion of exons 3–6 was reprogrammed from patient-derived urine cells following the reprogramming procedure as described previously^41,42^. Briefly, Dys3 urine cells were reprogrammed using the CytoTune iPS Reprogramming Kit (Life Technologies, Carlsbad, CA) containing Sendai virus (SeV) vectors with OSKM factors at the multiplicity of infection (MOI) of 1.5. After the expansion and thorough selection of reprogrammed iPSC, purified Dys3-iPSC clones were established for further characterizations and evaluations.

A separate wild-type iPSC line, HB53, was generously gifted by Dr. Ivor Benjamin and has been previously characterized^43^. HB53 was subjected to CRISPR gene targeting to introduce a mutation in the dystrophin gene which generated a dystrophin-deficient isogenic control iPSC line (DysC-iPSC) and untargeted cells were sub-cloned and served as an WT isogenic control iPSC line (WT2-iPSC).

For inert control fibroblast exosomes, we utilized a normal human dermal fibroblast cell line (Lonza, Basel, Switzerland) cultured in DMEM + 10% exosome depleted FBS.

### Characterization of reprogrammed Dys3-iPSC

Pluripotency of reprogrammed Dys3-iPSCs was confirmed through immunofluorescent staining of iPSCs with TRA-1-81 and Oct3/4 as performed previously (Supplemental Fig. 1)^41,42^. To evaluate non-integrating capacity of SeV vectors for efficient iPSC generation, Dys3 urine cells (UCs) and reprogrammed Dys3-iPSCs were characterized for pluripotency genes through RT-PCR analysis for exogenous reprogramming factors (Sev, Oct3/4, Klf4, cMyc) and endogenous pluripotent genes (Oct3/4, Sox2, and Nanog) as described previously (Supplemental Fig. 1)^41,42^.

### Embryoid body formation and three germ layer differentiation

To determine the pluripotent potentials of Dys3-iPSCs through spontaneous *in vitro* differentiation into three germ layers; ectoderm, mesoderm and endoderm, Dys3-iPSCs were cultured in suspension to form embryoid bodies (EBs) by hanging-drop protocol with STEMdiff™ APEL™ 2-LI Medium (StemCell Technologies, Vancouver, BC Canada) followed by adherent cell culturing on gelatin coated plates for 7days each. Dys3-iPSC derived spontaneously differentiated EBs were assessed for germ layer gene expression analysis by qRT-PCR for Nestin (ectoderm marker), Brachyury (mesoderm marker) and GATA4 (endoderm marker) (Supplemental Fig. 1). Primer sequences are listed in Table 1.

**Table 1:** Primers used for RT-PCR, qRT-PCR and genotyping PCR.

GAPDH	Forward	GTGGACCTGACCTGCCGTCT
	Reverse	GGAGGAGTGGGTGTCGCTGT
SeV (exogenous)	Forward	GGATCACTAGGTGATATCGAGC
	Reverse	ACCAGACAAGAGTTTAAGAGATATGTATC
Oct3/4 (exogenous)	Forward	CCCGAAAGAGAAAGCGAACCAG
	Reverse	AATGTATCGAAGGTGCTCAA
Klf4 (exogenous)	Forward	TTCCTGCATGCCAGAGGAGCCC
	Reverse	AATGTATCGAAGGTGCTCAA
cMyc (exogenous)	Forward	TAACTGACTAGCAGGCTTGTCG
	Reverse	TCCACATACAGTCCTGGATGATGATG
OCT4 (endogenous)	Forward	CAGTGCCCGAAACCCACAC
	Reverse	GGAGACCCAGCAGCCTCAAA
SOX2 (endogenous)	Forward	CAAGATGCACAACTCGGAGA
	Reverse	GTTCATGTGCGCGTAACTGT
NANOG (endogenous)	Forward	CGAAGAATAGCAATGGTGTGACG
	Reverse	CGAAGAATAGCAATGGTGTGACG
cTnT	Forward	AGCATCTATAACTTGGAGGCAGAG
	Reverse	TGGAGACTTTCTGGTTATCGTTG
GATA4	Forward	TCAGAAGGCAGAGAGTGTGTCA
	Reverse	CGTTGCACAGATAGTGACCCG
NKX2-5	Forward	ACAACTTCGTGAACTTCGGCG
	Reverse	GTGGACACTCCCGAGTTGCTCT
MYH6	Forward	CTCCTCCTACGCAACTGCCG
	Reverse	CGACACCGTCTGGAAGGATGA
MYH7	Forward	GGCAAGACAGTGACCGTGAAG
	Reverse	CGTAGCGATCCTTGAGGTTGTA
Nestin	Hs_NES_2_SG QuantiTect Primer Assay (Qiagen: QT01015301)	
Brachyury	Hs_T_1_SG QuantiTect Primer Assay (Qiagen: QT00062314)	
GATA4	Hs_GATA4_1_SG QuantiTect Primer Assay (Qiagen: QT00031997)	
DMD-exon 3	Forward	TCATCCATCATCTTCGGCAGATTAA
	Reverse	CAGGCCGTAGAGTATGCCAAATGAAAATCA
DMD-exon 4	Forward	TTGTCGGTCTCTCTGCTGGTCAGTG
	Reverse	CAAAGCCCTCACTCAAACATGAAGC
DMD-exon 6	Forward	CCACATGTAGGTCAAAAATGTAATGAA
	Reverse	GTCTCAGTAATCTTCTTACCTATGACTATGG
DMD-exon 12	Forward	GATAGTGGGCTTTACTTACATCCTTC
	Reverse	GAAAGCACGCAACATAAGATACACCT
DMD-exon 50	Forward	CACCAAATGGATTAAGATGTTCATGAAT
	Reverse	TCTCTCTCACCCAGTCATCACTTCATAG

### Karyotypic Analysis

Karyotyping was performed by Wisconsin Diagnostic Laboratories (Milwaukee, WI) to demonstrate euploidy of Dys3-iPSCs (Supplemental Fig. 1).

### Dystrophin Genotypic Analysis

To evaluate DMD gene mutations, genomic DNA from WT and Dys3-iPSCs was extracted with the QIAamp DNA Mini Kit (Qiagen, Valencia, CA) and DMD exons 3, 4, 6, 12 and 50 were amplified through PCR to screen the exon specific deletions in iPSC samples (Supplemental Fig. 1). The DMD exon specific primers used for the genotyping PCR are listed in Table 1.

### Gene editing in iPSCs

CRISPR target sites within exon 1 were identified using ZiFit Targeter version 4.2 (F: CACCGATACACTTTTCAAAAT GCTT and R: AAACAAGCATTTTGAAAAGT GTATC) and were cloned into pX330-U6-Chimeric-BB-CBh-hspCas9 (Addgene plasmid no 42230) as described previously^44^. An AAVS1 Safe Harbor TALE-Nuclease Kit including pAAVS1 Dual Promoter Donor Vector (GE602A-1) and the TALEN Vectors, pZTAAVS1 L1 TALEN Vector (GE601A-1) and pZT-AAVS1 R1 TALEN Vector (GE601A-1) was purchased from System Biosciences (Palo Alto, CA). HB53-iPSCs were transfected with or without all necessary components using 4D-Nucleofector (Lonza, Basel, Switzerland). Cells that underwent transfection by 4D Nucleofector but did not contain any targeting components were sub-cloned to generate the isogenic WT2-iPCS line. Following transfection with the targeting components, iPSCs were plated on mouse embryonic feeder cells for 9 days, and selected for puromycin resistance. Clones were picked, replated on Matrigel and sent out for *DMD* exon 1 sequencing (Retrogen, San Diego, CA). DysC-iPSCs were found to contain a 6 bp deletion in exon 1 and DysC-iCMs were identified as dystrophin deficient as shown by immunofluorescence (NCL-DysB, Leica Biosystems, Wetzlar, Germany) (Supplemental Fig. 2).

### iPSC culture

All iPSC lines were maintained on Matrigel (BD Biosciences, San Jose, CA, USA) in TeSR-e8 media (Stem Cell Technologies, BC, Canada). Cardiomyocytes were differentiated in RPMI media 1640 supplemented with B27 minus insulin (Differentiation media) and maintained in RPMI media 1640 supplemented with B27 plus insulin (Maintenance media; Thermo Fisher Scientific, Waltham, MA). All other reagents were obtained from Sigma (St. Louis, MO) unless otherwise specified.

### Cardiac differentiation

Wild-type and Dys-iPSCs were differentiated into cardiomyocytes (iCMs) as described previously^23^. In brief, iPSCs were seeded on to Matrigel-coated 12 well plates. On Day -1, Matrigel was overlaid in TeSR-E8™ media. On Day 0, Matrigel was overlaid in Differentiation Media with 9 μM CHIR (Selleck Chem, Houston, TX). On Day 1, Differentiation Media was replaced and 10 μM IWP-2 (StemGent, Cambridge, MA) was added on Day 3. On Day 5, Differentiation Media was replaced again and on Day 7, media was changed to Maintenance media. Cardiomyocytes contracting for 35 +/− 5 days were selected for experiments. Cardiac differentiations of WT1- and Dys1-iCMs have been characterized previously^23^. WT2-, Dys3- and DysC-iCMs were characterized by RT-PCR for markers cTNT, Nkx2.5, MYH6, MYH7, MLC2a and MLC2v, and by immunofluorescence for alpha-actinin (Abcam #ab72592, Cambridge, UK) and cTNT (Abcam #ab8295) as described previously (Supplemental Fig. 3)^23^.

### Characterization of iPSC-derived Cardiomyocytes

Cardiac gene expression analysis of iPSC-derived differentiated cardiomyocytes was performed as described previously^23^. Briefly, total RNA samples were extracted using the miRCURY RNA Isolation Kit - Cell and Plant (Exiqon, Denmark) and complementary DNA (cDNA) samples were synthesized using the iScript cDNA synthesis kit (Bio-Rad, Hercules, CA). The cDNA templates were amplified for the expression of cTnT, GATA4, Nkx2.5, MYH6, MYH7 and GAPDH genes through RT-PCR analysis. Gene expressions were detected through agarose gel electrophoresis (Supplemental Fig. 3) List of cardiac gene specific primers are listed in Table 1.

### Single Cell Dissociation

Single cells were dissociated using 0.05% Trypsin-EDTA (Thermo Fisher Scientific, Waltham, MA) for 5 minutes. Trypsin was inactivated with DMEM media (Thermo Fisher Scientific, Waltham, MA) with 10% fetal bovine serum. Cells were plated onto Matrigel-coated coverslips at a density of 50,000 cells per coverslip.

### Enhanced Green Fluorescent Protein Tagging of iPSC-derived cardiomyocytes

iPSC-derived cardiomyocytes were marked for live cell imaging as previously described^45^. In brief, 5 days after dissociation, iCMs were transduced with an NCX1-eGFP lentiviral construct^46^ encoding for enhanced green fluorescent protein (eGFP; MOI: 5.67 × 10^9^ IU/mL) under the control of the cardiac specific sodium-calcium exchanger 1 (NCX1) promoter. Living iCMs were identified by detecting green fluorescent cells, indicating NCX1-driven eGFP expression. Lentiviral vector assembly and titer determination was performed in the Lentiviral Core Facility at the Blood Research Institute of Wisconsin (Milwaukee, WI).

### Exosome isolation and treatment

Cells were plated 120,000 cells per well in a 12-well plate, conditioned media was harvested after 48 hours and exosomes were isolated with Total Exosome Isolation Reagent from Cell Culture Media (Thermo Fisher Scientific, Waltham, MA) according to the manufacturer’s protocol. The exosome pellet was resuspended in 100 μL of 0.2 μM filtered PBS. Exosomes isolated from a normal human adult dermal fibroblast cell line (Lonza, Basel, Switzerland) were used as an inert control.

### Confirmation of Exosome Surface Proteins

Surface proteins of isolated exosomes were confirmed using the Exo-Check™ Antibody Array (System Biosciences, Palo Alto, CA). A BCA protein array (Fisher Scientific, Hampton, NH) was used to determine exosomal protein concentration with 300 ug of protein being used for the arrays and the manufacturer’s protocol was followed. Protein blots were developed with the Bio-Rad CL Chemiluminescent developer (Bio-Rad, Hercules, CA) on a Bio-Rad Chemi-Doc (Bio-Rad, Hercules, CA) with 10 second exposure.

### Nanoparticle tracking analysis

For analysis of iCM-secreted exosomes, cells were plated in a 12-well plate, 120,000 cells per well. Media was harvested after 2 days, diluted 1:10 and perfused into the Nanosight (Malvern Instruments, Malvern, UK) for particle count and size analysis.

### Transmission electron microscopy

The ultrastructure of the exosomes was analyzed by the Medical College of Wisconsin Facility for Electron Microscopy (Milwaukee, WI, USA) using transmission electron microscopy. In brief, suspensions of exosomes were adsorbed onto on freshly ionized, 400 mesh formvar/carbon grids, washed once with distilled water and negatively stained with 2% aqueous Uranyl acetate. Exosome preparations were viewed in a Hitachi H600 transmission electron microscope and images recorded with a Hamamatsu ccd camera using AMT image capture software.

### Flow cytometry

Isolated exosomes were coupled to 4 μM latex beads (Thermo Fisher Scientific, Waltham, MA) and incubated with either CD81 (BD Biosciences, San Jose, CA, USA) or CD63 (Santa Cruz Biotech, Dallas, TX). Samples were run on a Becton Dickenson LSR II and data was analyzed with FlowJo v. 10.1.5 (Ashland, OR). Mouse IgG (Invitrogen, Carlsbad, CA) and rabbit IgG (Thermo Fisher Scientific, Waltham, MA) isotype controls were run in parallel with experimental samples. CD63 and CD81 expression was quantified by running bead + exosome and bead + exosome + 2′ antibody controls. Populations were gated by excluding false positive staining generated by controls.

### Exosome uptake into induced pluripotent stem cell derived cardiomyocytes

To assess exosome uptake by iCMs, exosomes from iCMs were labeled with PKH26 per the manufacturer’s protocol. Cells were incubated with PKH26 labeled exosomes (5 µL). After 2 hours, z-stack images of cells were taken using a Nikon A1-R confocal microscope (Nikon Instruments, Melville, NY).

### Stress-induced injury and treatment protocols

Cells on glass coverslips were exposed to 100 μM H_2_O_2_ in 10 mM deoxyglucose in RPMI minus glucose (Thermo Fisher Scientific, Waltham, MA) for 1 hour. Afterwards, cells were recovered in Maintenance media for 4 hours. This protocol mimics a transient metabolic/oxidative stress with recovery. Five µL of resuspended exosomes were added to iCMs for 2 hours prior to stress induction. In select experiments, iCMs were treated 30 minutes prior to exosome treatment with inhibitors (Invivogen, San Diego, CA): 5 µM SB203580 and 10 µM U0126.

### Superoxide and mitochondrial membrane potential staining

Cells were treated for 20 min with dihydroethidium (DHE, 10 µM) or tetramethylrhodamine ethyl ester (TMRE; 50 nM) to measure ROS levels and mitochondrial membrane potential (ΔΨ_m_) respectively. The fluorescent intensity of the ethidium derivative or TMRE was detected by a laser ex/em 518/605 nm or ex/em 540/595 nm, respectively. The changes in membrane potential were monitored by calculating relative TMRE fluorescence. Five ROIs were selected from the nucleus (DHE) or mitochondria (TMRE) of GFP-positive iCMs and measured for mean fluorescent intensity (ImageJ, Version 1.48 v, Java 1.6.0_65, National Institutes of Health, Bethesda, MD). Imaging conditions such as gain levels, frames per second and aperture size were held constant.

### Measuring mitochondrial permeability membrane transition pore (mPTP) opening

As described previously, cells were loaded with TMRE for 25 min at room temperature^45^. On laser-illumination, TMRE generates ROS within the mitochondria leading to mPTP opening and visualized by loss of the TMRE fluorescence. Time required to induce mPTP opening was determined from ΔΨ_m_ recordings. The peak signal value over the recorded region (50 µm^2^) was normalized as 100% and the lowest value as 0%. After normalization, the time required for a 50% decrease in signal was calculated and denoted as Time (s).

### Propidium iodide staining

Cell death was evaluated after a 24-hour recovery period, by labeling the cells with propidium iodide (PI, Thermo Fisher Scientific, Waltham, MA) per the manufacturer’s protocol. PI staining was quantified as a proportion of PI positive nuclei versus total nuclei.

### Immunofluorescence for Bax expression

iCMs were fixed with 4% paraformaldehyde (Alfa Aesar, Haverhill, MA) and stained with 1:500 Anti-Bax primary antibody (Abcam ab53154), and 1:200 goat anti-rabbit secondary antibody (Abcam ab97051) antibody. Mitochondria were co-stained with 500 nM Mitotracker (Thermo Fisher Scientific, Waltham, MA) for 30 min. at 37 °C. Cells were imaged with confocal microscopy and analyzed for co-localization in ImageJ. For a positive control, 1 mM staurosporine was added to cells for 1 hour at 37 °C.

### Detection of caspases 3 and 7

Caspase 3 and 7 were detected in iCMs with Image-IT LIVE Red Caspase-3 and -7 Detection Kit (Invitrogen, Carlsbad, CA) per the manufacturer’s protocol. Cells were imaged with confocal microscopy and % caspase 3/7 (+) cells versus total nuclei were quantified in ImageJ.

### Trypsinization of exosomes and protein array

Surface proteins were stripped from exosomes with 12.5 ng/μL trypsin (Thermo Fisher Scientific, Waltham, MA) and incubated for 3 hours at 37 °C. To confirm that surface exosomes were successfully removed, the trypsinized exosomes were subjected to an Exo-Check^TM^ Exosome Antibody Array as described above.

### ELISA assays for phospho-proteins

To assess phosphorylation levels of p38 MAPK and ERK1/2, cells were stimulated with exosomes for 0, 5, 10, 30 or 60 min, or with insulin as a positive control. Cells were washed with ice-cold PBS and rapidly lysed on ice using lysis buffer provided in the kit with protease and phosphatase inhibitors. p38 MAPK activation was assessed with p38 MAPK alpha (pT180/pY182) + total p38 MAPK alpha ELISA Kit (Abcam ab126453) per manufacturer’s instruction. ERK1/2 activation was measured with ERK 1/2 (Total/Phospho) InstantOne™ ELISA Kit (Thermo Fisher) per the manufacturer’s instruction. Absorbance was read at 450 nm on a microplate reader.

### Statistical analysis

Experimental results are presented as mean ± SEM. Student’s t-test and one-way ANOVAs were performed where appropriate (GraphPad version 6 for Windows). P-values ≤ 0.05 were considered statistically significant.

## Electronic supplementary material

Supplemental information

Supplemental video 1: PKH26 labeled exosomes are taken up by 2 hours in cardiomyocytes.

Supplemental video 2: U0126 does not block exosome uptake in cardiomyocytes.

## Data Availability

The datasets generated during and/or analyzed during the current study are available from the corresponding author on reasonable request.
