# Supplementary material for: Exosomes exert cardioprotection in dystrophin-deficient cardiomyocytes via ERK1/2-p38/MAPK signaling
Source: Sci Rep. 2018 Nov 8;8:16519. doi: 10.1038/s41598-018-34879-6 (PMC6224575; doi:10.1038/s41598-018-34879-6)
Supplement: Supplementary file 1 — Supplemental information [file 41598_2018_34879_MOESM1_ESM.docx]

**Supplemental information**

**Exosomes exert cardioprotection in dystrophin-deficient cardiomyocytes via ERK1/2-p38/MAPK signaling**

Melanie Gartz, MS, MHS^1,2^, Ashley Darlington, MD^1^, Muhammed Zeeshan Afzal, PhD,^1,2^ and Jennifer L. Strande, MD, PhD*^1,2,3^

**
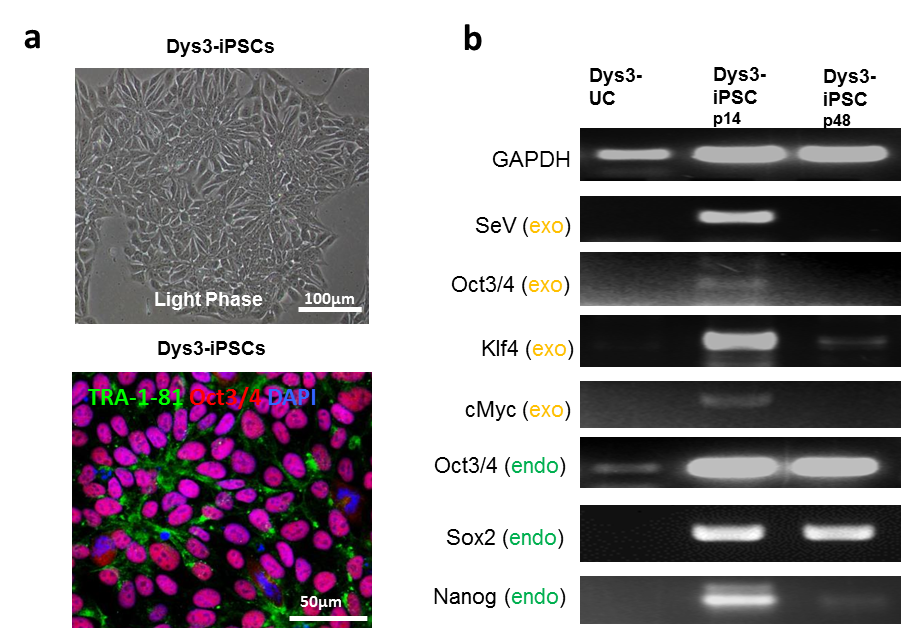
** **
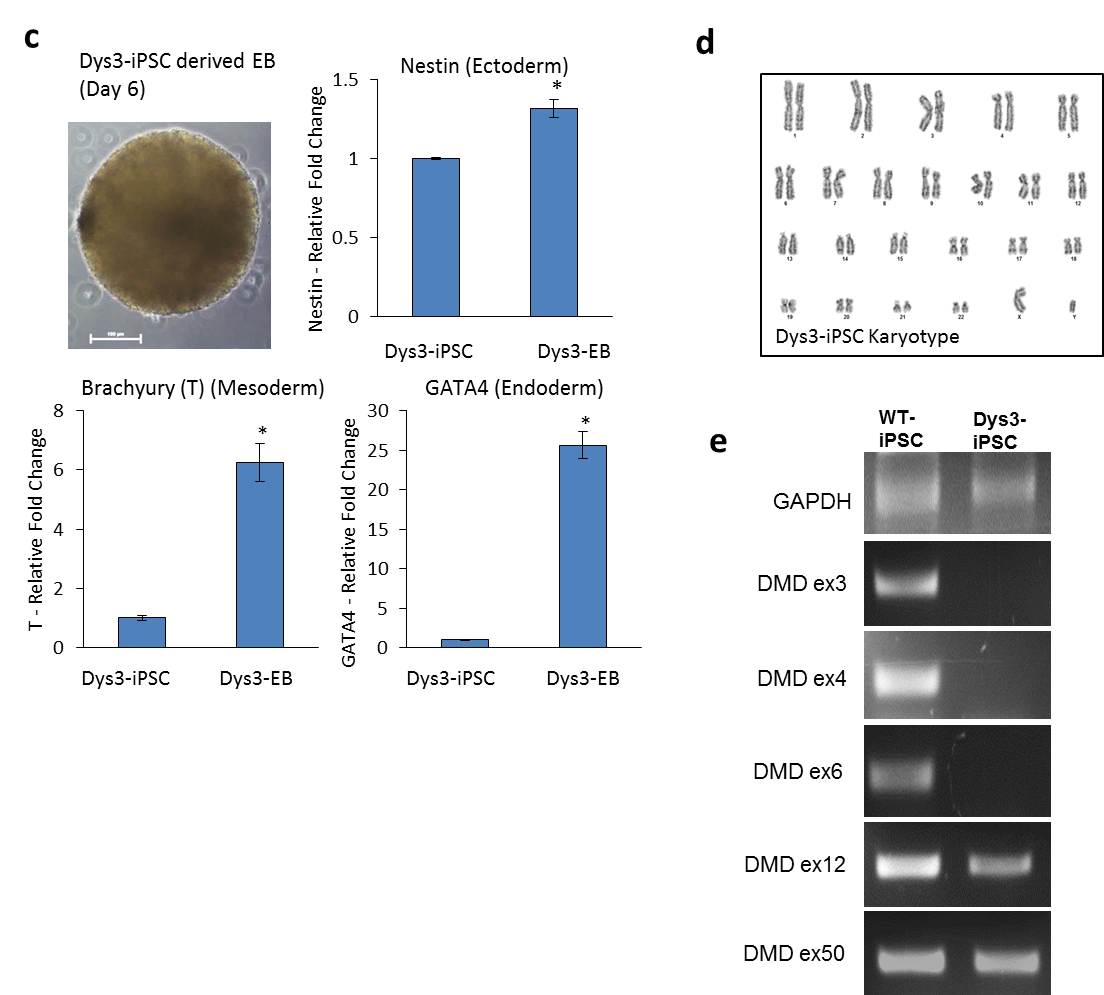
**

**Supplemental Figure 1: iPSC characterization of Dys3**. **a)** Immunofluorescence of Dys3-iPSCs for TRA-1-81 and Oct3/4. **b)** RT-PCR of Dys3 urine cells (UCs) and reprogrammed iPSCs for pluripotency markers. **c)** Dys3 embryoid body formation (EB) assay followed by qPCR of germ layer markers Nestin, Brachyury and GATA4. *p<0.05 EB vs. iPSC. **d)** Normal karyotype of Dys3-IPSCs. **e)** Genotyping of WT and Dys3-iPSCs for DMD exons 3, 4, 6, 12, and 50.

**
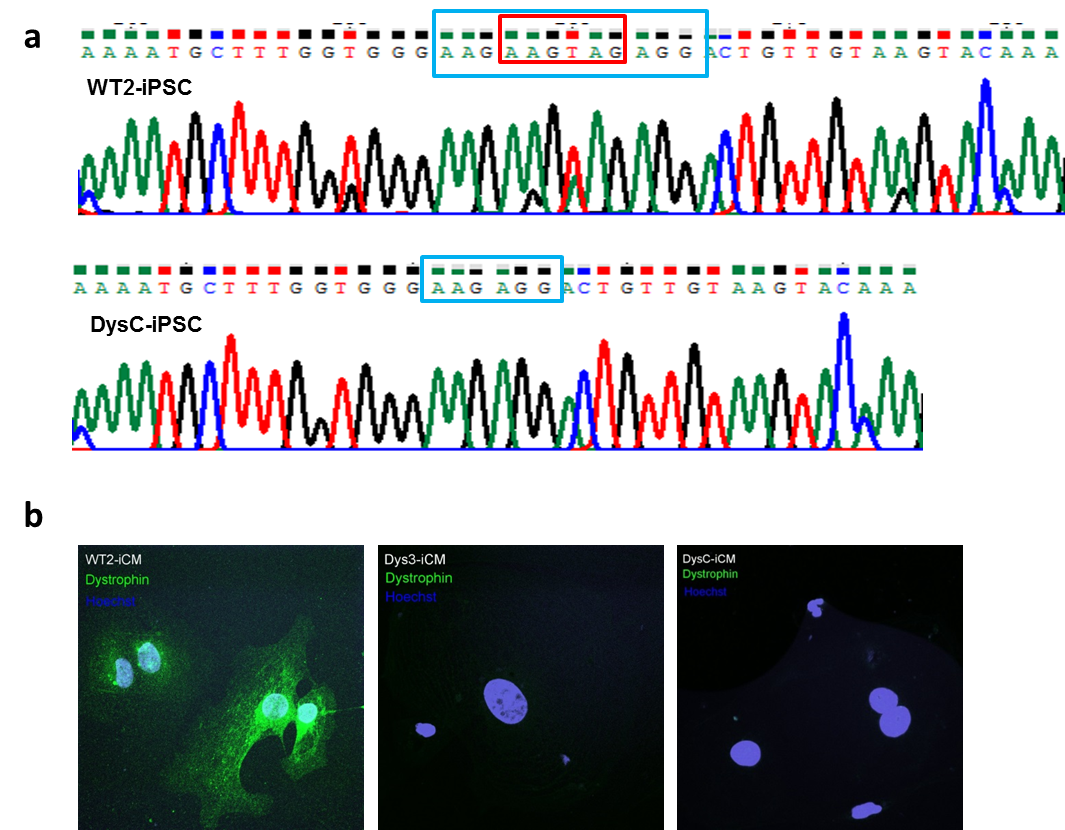
** **
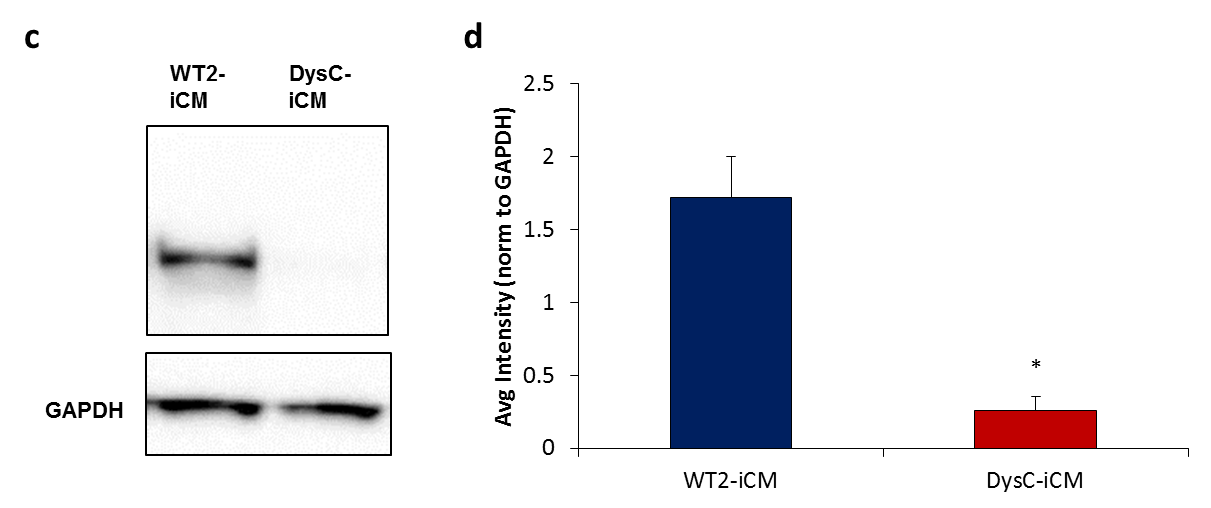
**

**Supplemental Figure 2: Characterization of dystrophin in iPSCs and iCMs.** **a)** Sequencing of CRISPR edited DysC-iPSCs shows a 6bp deletion in DMD exon 1. **b)** Immunofluorescence shows Dys3 and DysC-iCMs are negative for dystrophin staining (Abcam ab#15277). **c)** and **d)** Western blotting reveals diminished dystrophin expression in DysC-iCMs. *p<0.05 vs. WT-iCM.

**
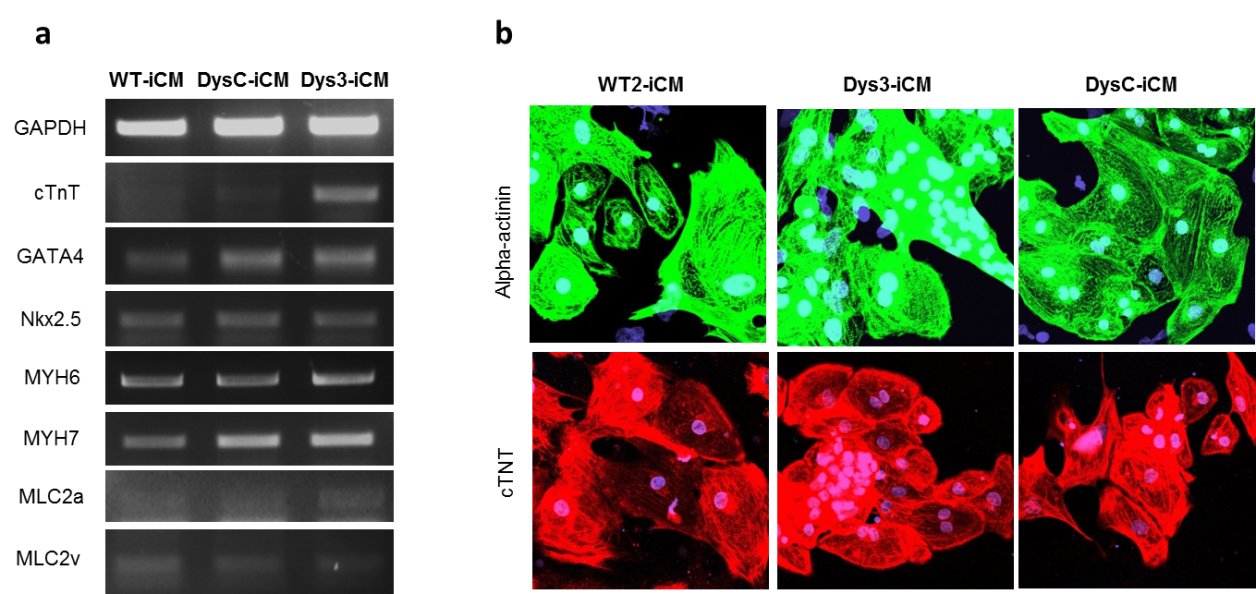
**

**Supplemental Figure 3: Characterization of iPSC-derived cardiomyocytes. a)** RT-PCR of cardiac differentiation markers in WT, DysC and Dys3-iCMs. **b)** Immunofluorescence of WT2, DysC and Dys3-iCMs for alpha-actinin and cTNT.

**
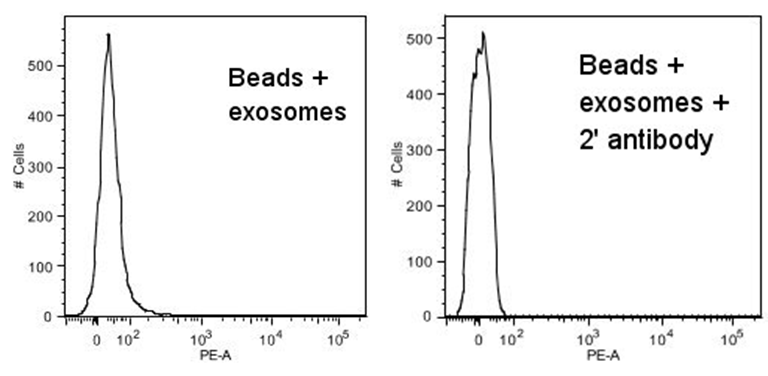
**

**Supplemental Figure 4: Isolated exosome flow cytometry controls.** Unstained control beads + exosomes and beads + exosomes + secondary antibody only were included in flow cytometry experiments to rule out false positive quantifications.

**
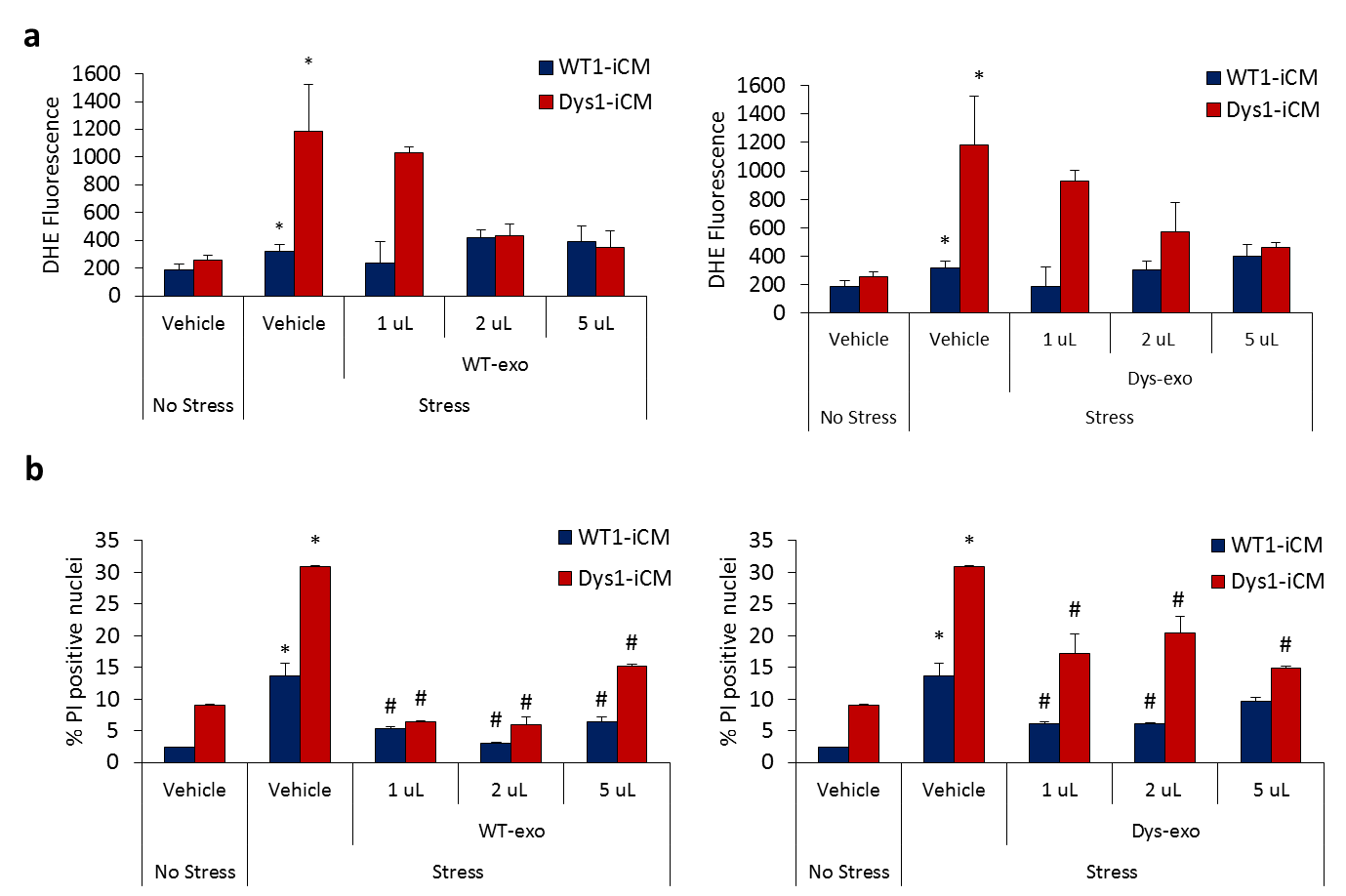
**

**Supplemental Figure 5: Optimizing exosome concentration for assays.** Assays examining **a)** ROS levels and **b)** cell death determined that 5 uL was the optimal volume of concentrated exosomes for use in subsequent assays. n=3/group. *p<0.05 vehicle stress vs. vehicle no stress, #p<0.05 exosome exposure vs. vehicle stress.

**
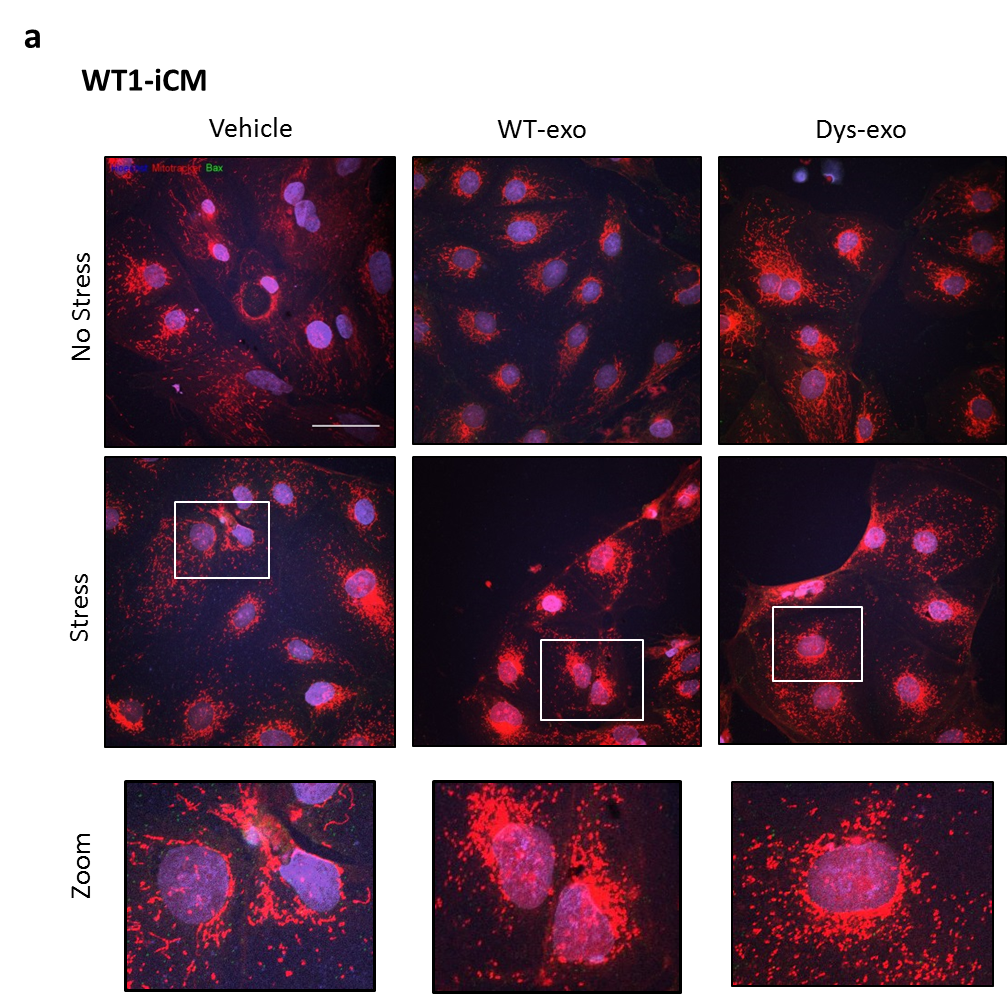
** **
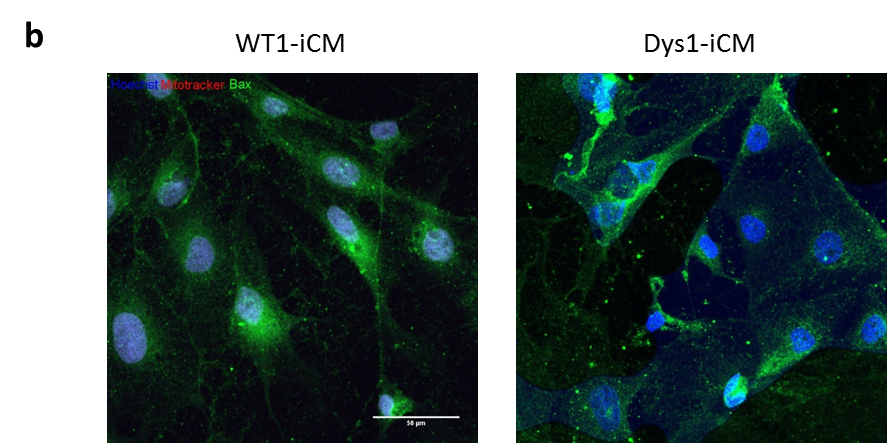
**

**Supplemental Figure 6: Bax expression analysis. a)** Vehicle + stress WT1-iCMs show rare Bax (green) staining overlaid with mitochondria (Mito tracker, red) and this is mitigated with 2 hr exosome exposure. n=3/group. **b)** Positive controls for Bax immunofluorescence. Apoptosis was stimulated with 100 nM Staurosporine for 1 hour at 37’C. All mitochondrial staining disappeared during apoptotic stimulation.

**
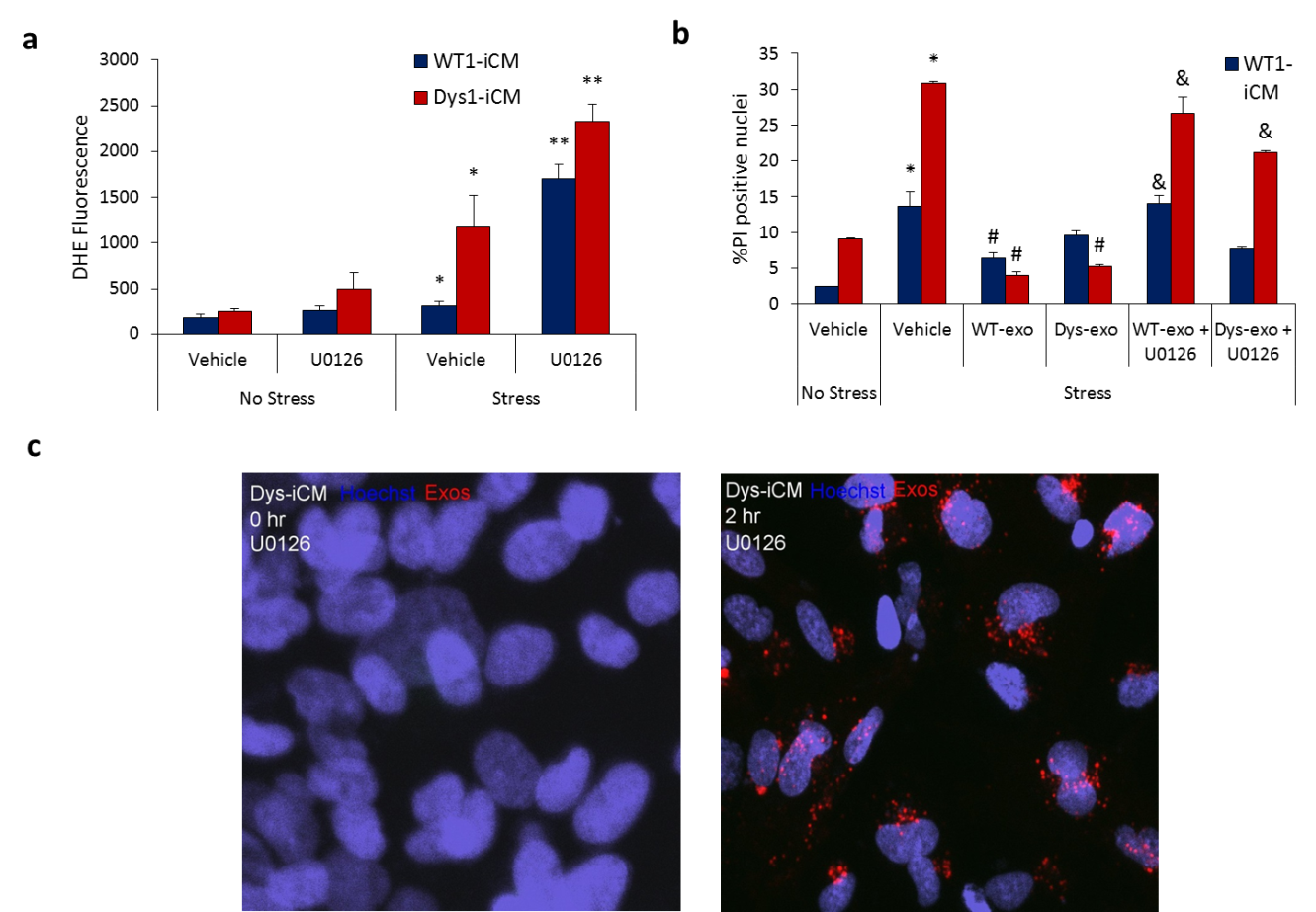
**

**Supplemental Figure 7: Examining the effects of U0126.** 10 um ERK1/2 inhibitor U0126 was used to assess **a)** ROS levels and b) cell death. **c)** At 2 hr, PKH26 labeled exosomes were observed to be taken up into Dys1-iCMs, indicating that U0126 did not prevent cardioprotection by inhibiting exosome uptake. n=3/group. *p<0.05 vehicle stress vs. vehicle no stress, **p<0.05 inhibitor vs. vehicle no stress.

**
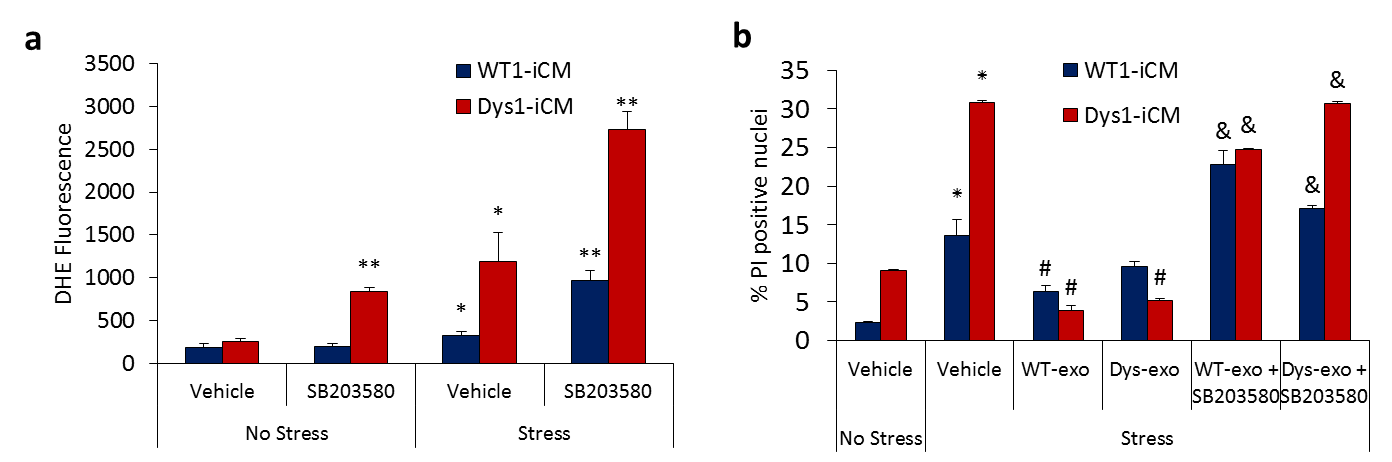
Supplemental Figure 8: Examining the effects of SB203580.** 10 uM SB203580 alone was tested in WT1 and Dys1-iCMs to evaluate **a)** ROS levels and **b)** cell death. n=3/group. *p<0.05 vehicle stress vs. vehicle no stress, **p<0.05 inhibitor vs. vehicle no stress.

**Supplemental video 1: PKH26 labeled exosomes are taken up by 2 hours in cardiomyocytes.** Exosomes were labeled with PKH26 and added to iCMs. Z-stack imaging was performed 2 hours after exosome addition, illustrating that exosomes were successfully taken up into cells. (Red= PKH26 stained exosomes, Green= NCX1-eGFP labeled cardiomyocytes, Blue= Hoechst).

**Supplemental video 2: U0126 does not block exosome uptake in cardiomyocytes.** 10 uM U0126 was added to cardiomyocytes 30 min prior to the addition of fluorescently-labeled exosomes. The concentration of U0126 used in experiments was not sufficient to block exosome uptake in cardiomyocytes. (Red= PKH26 stained exosomes, Green= NCX1-eGFP labeled cardiomyocytes, Blue= Hoechst).
